# Rap1 Can Bypass the FAK-Src-Paxillin Cascade to Induce Cell Spreading and Focal Adhesion Formation

**DOI:** 10.1371/journal.pone.0050072

**Published:** 2012-11-27

**Authors:** Sarah H. Ross, Emma Spanjaard, Anneke Post, Marjolein J. Vliem, Hendy Kristyanto, Johannes L. Bos, Johan de Rooij

**Affiliations:** 1 Department of Molecular Cancer Research, Centre for Biomedical Genetics and Cancer Genomics Centre, University Medical Center, Utrecht, The Netherlands; 2 Hubrecht Institute for Developmental Biology and Stem Cell Research, and University Medical Center, Utrecht, The Netherlands; King’s College London, United Kingdom

## Abstract

We developed new image analysis tools to analyse quantitatively the extracellular-matrix-dependent cell spreading process imaged by live-cell epifluorescence microscopy. Using these tools, we investigated cell spreading induced by activation of the small GTPase, Rap1. After replating and initial adhesion, unstimulated cells exhibited extensive protrusion and retraction as their spread area increased, and displayed an angular shape that was remodelled over time. In contrast, activation of endogenous Rap1, via 007-mediated stimulation of Epac1, induced protrusion along the entire cell periphery, resulting in a rounder spread surface, an accelerated spreading rate and an increased spread area compared to control cells. Whereas basal, anisotropic, spreading was completely dependent on Src activity, Rap1-induced spreading was refractory to Src inhibition. Under Src inhibited conditions, the characteristic Src-induced tyrosine phosphorylations of FAK and paxillin did not occur, but Rap1 could induce the formation of actomyosin-connected adhesions, which contained vinculin at levels comparable to that found in unperturbed focal adhesions. From these results, we conclude that Rap1 can induce cell adhesion and stimulate an accelerated rate of cell spreading through mechanisms that bypass the canonical FAK-Src-Paxillin signalling cascade.

## Introduction

The interaction between cells and extracellular matrix (ECM) proteins of the interstitial matrix and basement membrane is critical for the structural support of cells, as well as for supplying environmental cues that control the development, maintenance and integrity of tissues [1], [2]. Highlighting the importance of these processes is the vast array of diseases, both developmental and acquired, that derive from defects in extracellular matrix proteins or deregulated cell adhesion [1], [2], [3], [4], [5].

Cell adhesion and spreading is under the control of multiple signalling pathways which are derived both from the ECM constituents (outside-in signalling) as well as those originating from inside the cell (inside-out signalling) [6], [7], [8], [9], [10], [11], [12]. The integration of these signals controls the attachment and spreading of cells to a surface of ECM proteins, by regulating the assembly of focal adhesions (FAs). These large protein complexes consist of integrins, which facilitate both the attachment of cells and act as signalling receptors for the ECM protein ligand, as well as proteins, such as talin and vinculin, that initiate multiple links between integrins and the actin cytoskeleton [4], [10], [12], [13], [14].

In the canonical model of cell adhesion and spreading, outside-in adhesion signalling is initiated when integrins encounter their ECM ligands, and Src kinase is recruited to adhesion sites by its SH2 domain interacting with the autophosphorylation site of FAK (pY397) [10], [12]. Together, FAK and Src act as a signalling module to induce the phosphorylation of a number of focal adhesion proteins, including multiple sites on FAK itself, paxillin and p130Cas [10], [12], [13], [15]. These phospho-tyrosine residues act as docking sites for other proteins, which regulate the activities of the Rho family GTPases, Rac, Cdc42 and RhoA, to advance cell protrusion and spreading, and promote the link to the actin cytoskeleton [4], [10], [12], [14]. As the ECM-integrin-actin connection is formed, mechanical force develops across adhesions. Vinculin, in particular, is involved in strengthening integrin adhesions in response to force [16], [17], [18], [19], [20], [21].

The small GTPase, Rap1, is a known regulator of adhesion processes and can regulate integrins [22], [23], [24], [25], [26], [27], [28], [29], the actin cytoskeleton [30], [31], [32], [33], membrane protrusion [34] and the inactivation of RhoA [35], [36], [37], [38]. Furthermore, Rap1 activity has been linked to the control of talin, through its effector, Riam [39], [40], [41], [42], [43], to the inhibition of RhoA, via the effectors, Arap3 [35], [36], [44], [45], RA-RhoGAP/ARHGAP20 [46], [47], [48] and indirectly via the effector, Krit [37], [49], as well as to stimulation of Rac1, through regulation of Tiam1 and Vav2 [50]. Activation of Rap1 is spatially and temporally controlled by guanine nucleotide exchange factors (GEFs) which are themselves regulated by different stimuli. The GEF, C3G, acts downstream of Src [51], such that Rap1 may be activated in response to outside-in adhesion signalling [51], [52], [53]. However, Rap proteins can also function in inside-out cell adhesion pathways via GEFs regulated by second messengers, such as the cAMP-regulated Epac proteins and the calcium- and diacylglycerol-regulated CalDAG-GEFs [24], [25], [54], [55], [56].

Although implicated in several different aspects of cell-matrix interactions, the functional significance of Rap1 in cell adhesion processes is far less characterised than the roles of the GTPases, Rac1, Cdc42 and RhoA. Previously, we reported that when a suspension of A549-Epac1 cells was applied to an ECM-coated surface, activation of the Rap1 GTPase via Epac1 using the cAMP analogue, 8-pCPT-2′-O-Me-cAMP (also called 007), promoted focal adhesion formation, increased the spread area of cells and induced a round, rather than angular, cell morphology [57]. In this study, we performed live-cell imaging and developed new image analysis tools to enable a quantitative investigation into how activation of Rap1 can regulate the spreading process. Moreover, we have investigated how Rap1-induced spreading and FA formation relates to the canonical Src-mediated mechanism of cell spreading.

## Materials and Methods

### Cell Lines and Culture

The monoclonal Epac1-expressing A549-Epac1 cell line (derived from the A549 carcinoma (ATTC) and previously described in [34], [57]) and the derivative cell lines made in this study were cultured in RPMI supplemented with L-glutamine, antibiotics, and 10% fetal calf serum (FCS) (Gibco). Human umbilical vein endothelial cells (Lonza) were cultured using standard procedures [32].

Derivative A549-Epac1 cell lines, stably transfected with GFP-Lifeact or short hairpins, were made by a lentiviral delivery system. Lentiviruses were produced by transfection of 293T cells (ATCC) with SIN-inactivated virus constructs. A549-Epac1 cells were plated in full medium in a 6-well plate overnight and then transduced with GFP-Lifeact [58] or short hairpin virus supernatants in the presence of 4 µg/ml polybrene for 24 hours before returning the cells to full growth medium. GFP-Lifeact-expressing cells were selected by fluorescence-activated cell sorting (FACS) by their GFP expression levels, while cells expressing the short hairpins were selected by adding puromycin to the culture medium at a concentration of 2 µg/ml.

### Reagents and Antibodies

8-pCPT-2′-O-Me-cAMP (007) was obtained from BioLog Life Sciences Institute. PP2 was from Torcis Bioscience and PF573228 and Y27632 were from Sigma-Aldrich.

Antibodies were from BD Biosciences (paxillin, FAK, Rock I and Rock II), Chemicon (α-tubulin), Sigma-Aldrich (vinculin), Cell Signaling Technology (β-actin, phospho-FAK Y576/577, phospho-FAK Y925, phospho-paxillin Y118, phospho-Src Y416), Invitrogen (phospho-FAK Y397), GeneTex (phospho-FAK Y861) and Millipore (GAPDH). The integrin inhibiting antibodies AIIB2 (anti-β1) and GOH3 (anti-α6) were derived from the hybridoma cell lines (antibody-containing tissue culture supernatant was a gift from A. Sonnenberg, The Netherlands Cancer Institute, Amsterdam). The Pelicluster CD61 antibody (anti-β3) was from Sanquin.

Control and FAK-targeting short hairpin lentiviral MISSION vectors from the TRC1 library were obtained from Sigma-Aldrich. ON-targetplus SMARTpool siRNA oligos were obtained from Dharmacon and were used to deplete Rap1A, Rap1B, Rock I, Rock II and FAK.

Fibronectin was purified from human plasma as described previously [59].

### DNA Constructs

The pRRL LifeAct-pEGF construct for the production of GFP-Lifeact lentiviruses was a gift from O. Pertz (University of Basel, Switzerland).

### Cell Transfection

For knockdown experiments, A549-Epac1 cells were seeded sparsely and transfected straight away with 50 nM siRNA oligos (Dharmacon) using HiPerFect (Quiagen) according to the manufacturer’s protocol. Transfected cells were left for 48 hours before being analysed further.

### Short-term Adhesion Assays

Short-term adhesion assays were performed as described previously [57]. A549-Epac1 cells were trypsinised, washed in RPMI containing 10% FCS, and kept in suspension for 1.5 hours in RPMI containing 0.5% FCS, glutamine, antibiotics, and 20 mM Hepes, pH 7.4, at 37°C. 48-well plates were coated with fibronectin overnight at 4°C, and blocked with heat-denatured bovine serum albumin (BSA) for 1 hour at 37°C. Cells were plated into the fibronectin-coated wells and allowed to adhere for 30 minutes at 37°C in the presence or absence of 100 µM 007. All conditions were performed in quadruplicate. After 30 minutes, non-adherent cells were removed and adherent cells were washed once with pre-warmed phosphate-buffered saline (PBS). Cells were lysed in alkaline phosphatase buffer (0.4% Triton X-100, 50 mM sodium citrate, and 10 mg/ml phosphatase substrate (Sigma-Aldrich)). The total number of cells adhering was determined by phosphatase assay [60]. Aliquots of the cell suspension added to each well were taken, pelleted by centrifugation and then lysed to determine the total number of cells added per well. The adhesion of cells to wells blocked with heat inactivated BSA but not coated with fibronectin was taken as background, and subtracted from the readouts for basal and 007-induced adhesion. The adhesion over 30 minutes was expressed as a fraction of the total cells added to the wells.

Inhibiting antibodies, Pelicluster CD61 (used at a 1∶100 dilution), AIIB2 (used at a 1∶10 dilution) and GOH3 (used at a 1∶10 dilution) were added to cells just prior to plating out onto fibronectin. In controls, 1% FCS in DMEM was added in a 1∶5 dilution and 10 mg/ml BSA was added at a 1∶100 dilution to take into account any effects of the storage buffer of the antibodies.

### Spreading Assays

In spreading assays, either for live-cell imaging or for fixed immunofluorescence assays, cells were trypsinised using a 1∶4 dilution of the trypsin stock, washed once with the appropriate media with 10% FCS and then kept in suspension for up to 1.5 hours at 37°C in media containing 0.5% FCS, glutamine, antibiotics and 20 mM Hepes. Cells for immunofluorescence were maintained in RPMI, while cells used for live-cell imaging were kept in suspension in Leibovitz’s L15 medium (Gibco). For spreading assays with human umbilical vein endothelial cells, the cells were kept in full growth media. During the time in suspension, cells were incubated with inhibitors (20 µM PP2 or 1 µM PF573228) as required. Following recovery, cells were plated onto glass which had been coated with fibronectin overnight at 4°C at a density of 1.25×10^4^ cells/cm^2^. For immunofluorescence, cells were applied to coverslips in 24-well plates or, for live-cell imaging experiments, into LabTekII 8-chambered slides (Nalge Nunc International).

For live-cell imaging, cells were plated out in the presence or absence of 100 µM 007 and imaged from approximately 30 minutes after plating, for the following 3 hours. Cells did vary in the extent to which they had already spread at the commencement of imaging, but cells were always captured during the time when the spreading rate over the first 30 minutes of imaging was linear. Imaging was managed using Metamorph software (Molecular Devices), with images being captured every 5 minutes using a Zeiss Axiovert 200 M microscope in a climate-controlled incubator. Stage positions were controlled using a Zeiss MCU 28 robotic stage. Images of GFP-Lifeact-expressing cells were collected using a Lambda DG-4 Ultra High Speed Wavelength Switcher (Sutter Instruments) as a light source and a Coolsnap HQ CCD camera (Photometrics) through a Zeiss Fluar 20×objective or a Zeiss PLAN NeoFluar 63×objective. For each experiment, at least five different stage positions were acquired for each spreading condition.

In immunofluorescence assays, cells were allowed to adhere and spread for 3 hours in the presence or absence of 100 µM 007. After 3 hours, the cells on the coverslips were fixed in cytoskeletal buffer (0.5% Triton X-100, 10 mM Pipes pH 7, 50 mM KCl, 2 mM CaCl_2_, 2 mM MgCl_2_, 300 mM sucrose) with 4% (v/v) paraformaldehyde for 15 minutes. Coverslips were blocked overnight in 2% (w/v) BSA in Tris-buffered saline (TBS) at 4°C. Focal adhesion proteins were detected using the appropriate antibodies and Alexa 568- or Alexa 488- coupled secondary antibodies. Actin was visualised using phalloidin coupled to Alexa 488 (Invitrogen). Immunofluorescence images were acquired using a Zeiss Axioskop 2 microscope fitted with a Zeiss Axiocam CCD camera and 40×and 100×Plan APO objective lenses.

### Automated Image Analysis and Quantification

For automated analysis, a detection and tracking algorithm was implemented in MatLabR2008a (Mathworks, Inc.). Cells were detected in images using local watershed segmentation upon smoothing. To ensure that all data was collected for the same, single cell over the course of a time-lapse movie, images captured through the 63×objective lens were cropped so that only one cell was visible in each frame. To maximise the number of cells for statistical analysis, we used the 20×objective to image cells which had been transfected with siRNA or cells where PP2 was to be added post-spreading. In images taken through the 20×objective, multiple single cells were analysed for each frame. Individual cells were tracked in time, linking cells from one frame to the next using the overlap of detected objects between consecutive frames. In situations where the detection of a cell was lost between frames, and, therefore, an overlap could not be detected, the object was linked with the five subsequent time-frames so that the tracking of the object could be continued as long as possible. To remove erroneously detected background structures, objects with an average intensity similar to the average intensity of the whole image were discarded. All tracks were validated manually. The size of the spread area of a cell was evaluated by determining the number of pixels within the region of detection, and plotted over time to analyse the spreading kinetics. In 20×images, the spreading areas of all the tracked objects were averaged to obtain the mean spread area of cells in the entire field of view. In a single experiment, the final result for a particular spreading condition was the average of five different 20×fields of view. The average spreading rate was calculated by determining the gradient (m) of the increase in spread area per hour during the first 30 minutes of imaging. Due to differences in imaging conditions, the m values calculated from images collected using the 20×objective lens are not directly comparable to those obtained from images gathered by the 63×objective lens. Statistical analyses on time course data were performed by two-way ANOVA with replication.

Protruding and retracting areas in the 63×images were determined by subtracting the segmented image at a particular time point from the subsequent segmented image in the time-lapse movie to produce an image showing regions gained, lost or unchanged between the two frames. In this image, pixels that were unchanged between frames had a value of 0, pixels that were lost in the subsequent frame (retractions) had a value of −1, while pixels that appeared in the succeeding frame (protrusions) had a value of +1 (Figure S1) and the total number of pixels with value −1 or +1 was determined. Total area of protrusion and retraction per image was then calculated as percentage of the total cell area in the second frame used in the image calculation. The circularity (circle ratio) of a cell was calculated by quantifying the ratio between the cell area and the area of the circle with a radius measuring the same distance as that between the cell centre and the most distant pixel detected on the edge of the cell at that time point (Figure S1). For the quantification of the changes to spread area shape over time, at each time point, the standard deviation of the distribution of the distances from the cell centre to every pixel on the cell edge was determined and normalised to the mean distance to correct for differences in cell size (Figure S1). The normalised standard deviation (σ_n_) was calculated using the standard equation. The variance at time t was subtracted from variance at time t+1 and the average difference of the variance (Δσ_n_) between consecutive time-lapse frames for all analysed cells was determined.

### Quantification of Cell Spread Area in Immunofluorescence Assays

ImageJ (NIH) was used to quantify cell spreading of fixed and stained cells as described previously [57]. Briefly, for each spreading condition, images of at least 30 cells taken from a minimum of 3 different fields of view were captured through the 40×objective. The average cell spread area, in pixels, for each condition was calculated per data set. Data for these individual experiments were then standardised to a value determined by averaging the spread area of control cells for all the data sets analysed. Statistical analysis was performed by paired student’s t-tests.

### Quantification of Focal Adhesions in Immunofluorescence Assays

To analyse the composition of focal adhesions under different conditions, the fluorescent intensity of immunolabelled components in fixed cells was measured. In these assays, the levels of focal adhesion-localised paxillin and vinculin were analysed in single immunostainings with a phalloidin counter-stain, while the levels of phospho-FAK and phospho-paxillin were analysed by dual immunostaining with unphosphorylated paxillin. In order to permit comparison between the intensity levels of staining, the same exposure conditions were used to capture all images for a particular staining within an experiment. The secondary antibodies did not stain components of focal adhesions independently of the primary antibody. Focal adhesions were detected, based on the paxillin or vinculin signal, using local watershed segmentation. In the dual stainings, the objects detected by the paxillin staining were used as mask for detecting the region in which to analyse the phospho-focal adhesion protein. The cell outline was determined by thresholding the phalloidin staining or by thresholding the paxillin signal in the dual focal adhesion stained cells. All measured focal adhesions were within the designated cell area and outside a user specified area, which allowed the false detection of nuclear spots to be excluded. For each image, the average fluorescence intensity outside the cell area was subtracted from the image to remove camera and other noise. The average fluorescence intensity detected within the cell area, excluding the regions detected as focal adhesions, was then taken as the cellular background. The total intensity of all the detected focal adhesions in the cell was determined and normalised to the cellular background. For the paxillin and vinculin single stainings, in each experiment, the average intensity of all cells analysed per condition was determined. Data from multiple experiments were standardised to the value obtained by averaging the intensity of 007-only treated cells from all the data sets gathered. The vinculin composition under different conditions was analysed by determining the ratio between the normalised vinculin and normalised paxillin levels. For the dual stained adhesions, the ratio between the normalised paxillin and the normalised phosphorylated adhesion protein was calculated. Paired student’s t-tests were used to perform statistical analyses.

### Isolation and Analysis of Rap-GTP

Cells were transfected with siRNA for 48 hours and then stimulated with mock or 100 µM 007 for 15 minutes. Cells were lysed in Ral buffer (50 mM Tris.HCl pH 7.5, 200 mM NaCl, 2 mM MgCl_2_, 1% (v/v) NP40, 10% (v/v) glycerol, 1 mM PMSF, 1 µM leupeptin, 0.1 µM aprotinin), and the lysates were pre-cleared by centrifugation. The Rap-GTP was captured using the RalGDS-Rap-binding domain, pre-bound to glutathione agarose, over 45 minutes as described previously [61]. Endogenous Rap1 was detected by western blotting.

### Western Blotting to Analyse Activation of the Src-signalling Pathway

As for spreading assays, cells were trypsinised, washed once with the appropriate media with 10% FCS and then kept in suspension for 1.5 hours at 37°C in media containing 0.5% FCS, glutamine, antibiotics and 20 mM Hepes in the presence or absence of the appropriate inhibitors. Cells were allowed to adhere in the presence or absence of 100 µM 007 for 30 minutes or 3 hours and lysed using Laemmli sample buffer. Depletion of proteins by siRNA was determined by lysing cells transfected for 48 hours in Laemmli buffer.

Protein samples were separated using SDS-PAGE and transferred to PVDF membranes (Immobilon). The membranes were blocked for 1 hour using 2% BSA and then probed with the appropriate primary antibody. The antibodies were detected by anti-mouse or anti-rabbit antibodies conjugated to horseradish peroxidase, and proteins were detected by ECL.

## Results

### Activation of the Epac1-Rap Pathway Alters the Spreading Dynamics and Morphology of Cells

**Figure 1:**
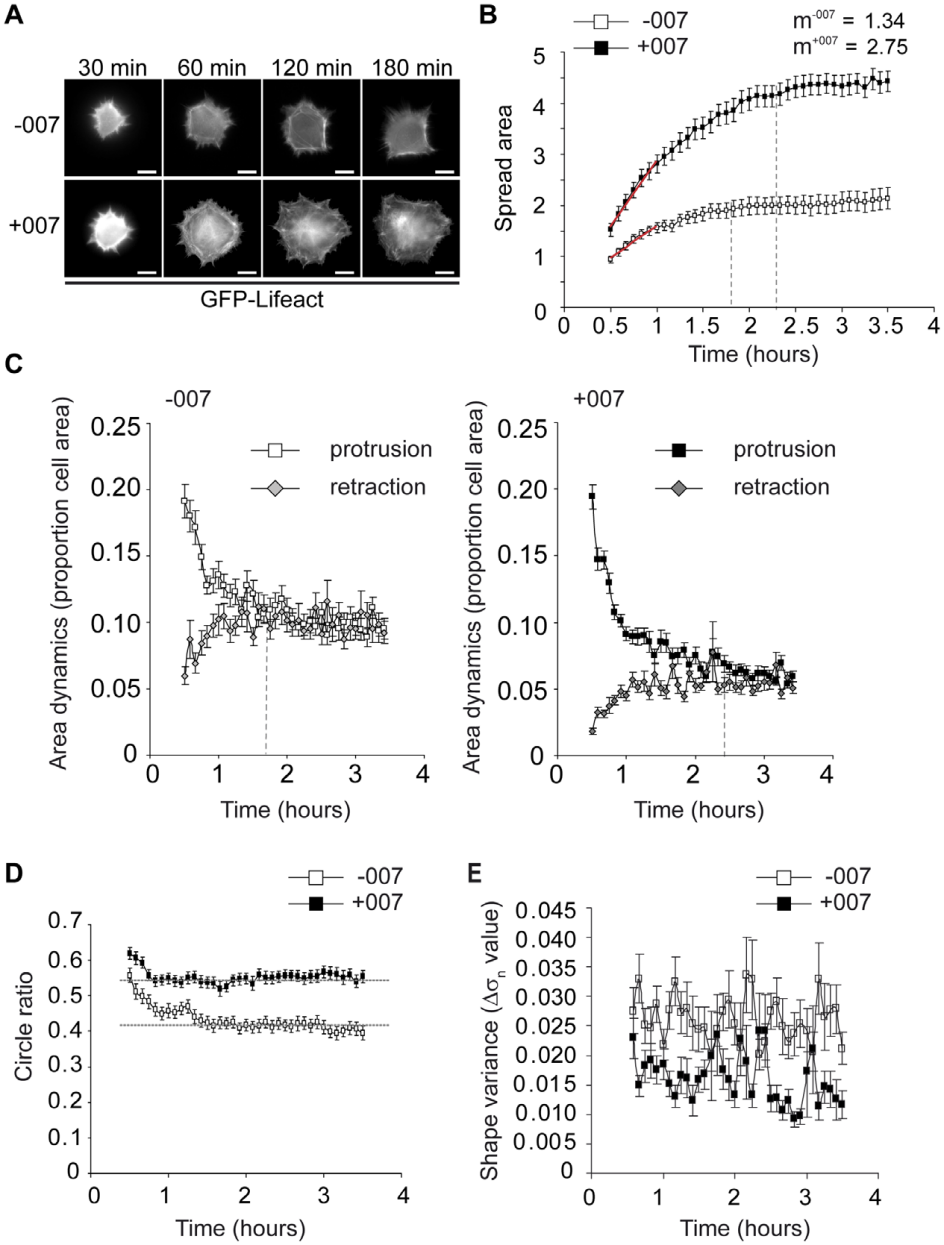
Live-cell imaging of 007-induced adhesion and spreading. A549-Epac1 cells expressing GFP-Lifeact were trypsinised and allowed to roll for 1 hour before plating out on fibronectin with or without 100 µM 007. Images of cells were captured through the 63×objective every 5 minutes from 30 minutes after plating, with representative images shown in (A). The scale bars represent 10 µm. Altogether, 54 cells without 007 and 41 cells with 007 from 10 separate spreading experiments were quantified. The spread area at each frame was determined in pixels and standardised to the average area of all the first frames of the basal (−007) spreading cells (B). The spreading rate over the first 30 minutes of imaging was calculated using the gradient (m) of the red lines show on each graph and is presented in spread area per hour. Dashed vertical lines indicate the time at which cells approached their maximal area. Area dynamics (C) were determined by measuring the gain or loss of spread area between consecutive frames, and presented as a proportion of the spread area of the second frame. The dashed vertical lines show the time at which the area of protrusion equalled the area of retraction. Cell morphology was analysed by the circle ratio (D), which was calculated by dividing the area of the cell at each time point by the area of a circle which encompassed the outer-most tips of the detected cell edges. Changes in the shape of the spread area of cells were calculated by determining the normalised variance (σ_n_ value) of the distances from the cell centre to each peripheral point of the cell. Then, the change in the variance (Δσ_n_ value) between consecutive time points was calculated (E). For all graphs shown, each plotted point is the mean of all cells measured ± the standard error of the mean.

To investigate how activation of the Epac1-Rap1 signalling pathway led to the large and round cell spreading phenotype in A549-Epac1 cells [57], we performed live-cell imaging and examined the differences in the morphology and in the dynamics of spreading over time in the absence or presence of the Epac1 activator, 007 (Figure 1 and Movie S1 and Movie S2). Cells were replated onto fibronectin without or with 100 µM 007 and imaged from approximately 30 minutes after their first contact with fibronectin for the next 3 hours (Figure 1A and Movie S1 and Movie S2). We then developed custom automated image analysis software in order to quantify and to characterise cell spreading in the presence and absence of 007 (described in Materials and Methods, Figure S1, Movie S3 and Movie S4).

In the presence and absence of 007, cells spread rapidly over the first hour after plating, and in general, cells stimulated with 007 were already more spread than control cells at the commencement of imaging (Figure 1A and 1B). Over the first 30 minutes of imaging, cells with 007 spread at twice the rate of cells without 007 (Figure 1B). After the period of rapid spreading, the cells continued to increase their spread area at a lower rate, until reaching a steady state of total contact with the matrix (Figure 1B). In cells without 007, this maximum spread area was reached after approximately 110 minutes of contact with the ECM, while cells treated with 007 took around 140 minutes to approach their maximal spread area. Therefore, the increase in the spread area of A549-Epac1 cells induced by 007 that we observed after 3 hours of spreading in a fixed time point assay [57] is the result of both a prolonged and a faster rate of cell spreading.

The time-lapse imaging revealed that under basal conditions, cells displayed an anisotropic mode of spreading with unequal protrusion along the cell periphery. Moreover, the spreading was accompanied by remodelling of the spread surface, and cells displayed periods of retraction and protrusion as they increased their coverage of the ECM and as they maintained their maximal spread area (Figure 1A and Movie S1). To analyse the spreading process in further detail, we determined the areas of the spread surface of cells which were gained (protrusions) or lost (retractions) between consecutive imaging frames and related this to the spread area which resulted from these changes (Figure 1C). To initiate spreading, cells in the presence or absence of 007 showed greater protrusion than retraction, consistent with the cells making rapid and persistent spreading contacts with the ECM. Noticeably, cells treated with 007 retracted a smaller proportion of their spread area than basal spreading cells, indicating that retraction was decreased by 007. As cells approached their maximum spread area, protrusion activity decreased (Figure 1C), and an equilibrium between the areas of protrusion and retraction was reached. At this point, cells treated with 007 remodelled a smaller proportion of their spread area, compared to basal cells, whilst maintaining their maximum spread area (Figure 1C). These data imply that 007 inhibits cell retraction and stabilises the contacts between cells and their environment.

The round, rather than angular, mode of cell spreading induced by 007 was established by the initiation of more persistent protrusion sites around the entire periphery of the cell than in basal conditions, and, hence, cells exhibited isotropic cell spreading (Figure 1A and Movie S1 and Movie S2). To analyse the form of cells over time further, we calculated the circle ratio of the cell at each time point by determining what proportion of a circle, encompassing the outer-most tips of the detected cell edges, was occupied by the area of the cell (Figure 1D). Thus, a higher circle ratio is indicative of a round cellular shape. Cells spreading without 007 had a lower circle ratio compared to cells spreading with 007, demonstrating that under basal conditions, cells were, indeed, more angular throughout the time course of spreading (Figure 1D). To evaluate cellular form and morphology dynamics further, we devised a method to quantify the changes in shape as the difference of the variance (Δσ_n_) of the distance from the cell centre to all points on the cell periphery between consecutive time-lapse frames (Figure 1E). Cells spreading without 007 displayed a higher value of Δσ_n_ than 007-treated cells, reflecting the dynamic shape changes of basal spreading cells and the more consistent shape of the 007-stimulated cells as observed in the time-lapse-movies (Movie S1 and Movie S2 and Figure 1E). This quantitative live-cell imaging shows that 007 promotes a faster rate of cell spreading by promoting protrusion along the entire cell edge, while reducing the retraction activity exhibited by unstimulated cells as they spread.

### Rap1A and Rap1B Contribute to Basal Spreading of Cells and are Responsible for the 007 Effects

**Figure 2:**
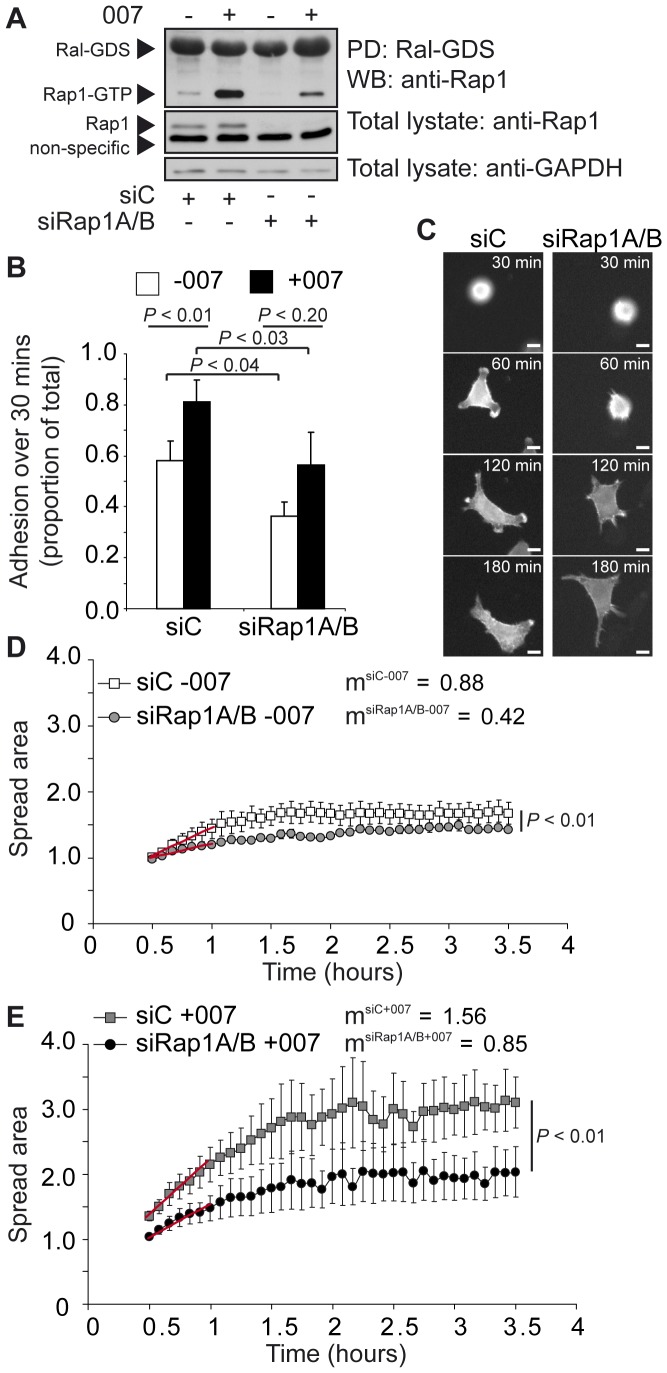
Effects of depleting Rap1A and Rap1B on cell spreading. A549-Epac1 cells were transfected with scrambled siRNA (siC) or siRNA against Rap1A and Rap1B together (siRap1A/B) for 48 hours before further analysis. In (A), cells were exposed to mock or 100 µM 007 for 15 minutes. Pull down of Rap1-GTP, by the RalGDS-Rap-binding domain, and knockdown of Rap1A/B were detected by western blot. The depletion of Rap1A and Rap1B is representative of the knockdown achieved in cells used for the adhesion and spreading analyses. In (B), siRNA transfected cells were replated with or without 100 µM 007 and cell adhesion after 30 minutes was determined by alkaline phosphatase activity. Adhesion is expressed as a proportion of the total cells added per well and the graph shows the mean adhesion for three experiments ± the standard error of the means. The *P* value was determined by a paired student’s t-test. For spreading analysis, cells treated with siRNA were plated on fibronectin and images were captured every 5 minutes using the 20×objective. Representative time-lapse images of basal spreading cells are shown in (C). The scale bars represent 10 µm. Cell spreading was quantified for time-lapse movies under basal conditions (D) and in the presence of 100 µM 007 (E). The spread area for each knockdown condition was calculated by standardising all measurements to the average size of siC cells without 007 at the first imaging time point. The graphs show the mean of the spread area for three knockdown experiments ± the standard error of the means. The rate of increase in the spread area of cells over the first 30 minutes of imaging, shown with red lines on the graphs, was determined by calculating the gradient (m) of the lines (calculated as spread area per hour) and is presented alongside the key. The *P* values were calculated using a two-way ANOVA with replication.

In order to confirm that the effects of 007 on cell spreading were mediated by Rap1 activity, and to investigate whether the activity of Rap1 was required for basal spreading, we transfected cells with siRNA targeting both Rap1A and Rap1B and investigated the effects on cell spreading. The reduction of Rap1A/B was sufficient to decrease basal and 007-induced Rap1-GTP levels (Figure 2A). As we previously observed with depletion of Rap1A alone [57], depletion of Rap1A/B reduced the adhesion of cells over 30 minutes (Figure 2B). However, depletion of Rap1A/B neither altered the angularity of cells nor prevented the membrane protrusion and retraction activity during basal spreading (Figure 2C and Movie S5 and Movie S6). Quantification of time-lapse movies of Rap1A/B-depleted cells spreading under basal conditions showed that at the commencement of imaging, these cells had approximately the same spread area as control cells transfected with a scrambled, control, siRNA and could initiate spreading (Figure 2C and 2D and Movie S5 and Movie S6). However, over the first 30 minutes of spreading, cells depleted of Rap1A/B spread with a rate which was approximately half of the spreading rate observed with control cells, and showed a 20% reduction to the maximal spread area (Figure 2D). Depletion of Rap1A/B significantly decreased the spreading response induced by 007 (Figure 2E and Movie S7 and Movie S8). However, compared to basal conditions, a small induction of initial spreading rate and maximum spreading was observed in cells depleted of Rap1A/B in response to 007. This is most likely explained by the incomplete depletion of Rap1 by the siRNAs, and the increase in GTP-loading of this residual Rap1 by 007 (Figure 2A). From these results, we conclude that Rap1A/B is required for 007-induced cell spreading, and controls the basal rate of cell spreading and contributes to the maximum spread area which cells can sustain.

### 007 Bypasses the Requirement of Src in Cell Adhesion, Cell Spreading and FA Formation

**Figure 3:**
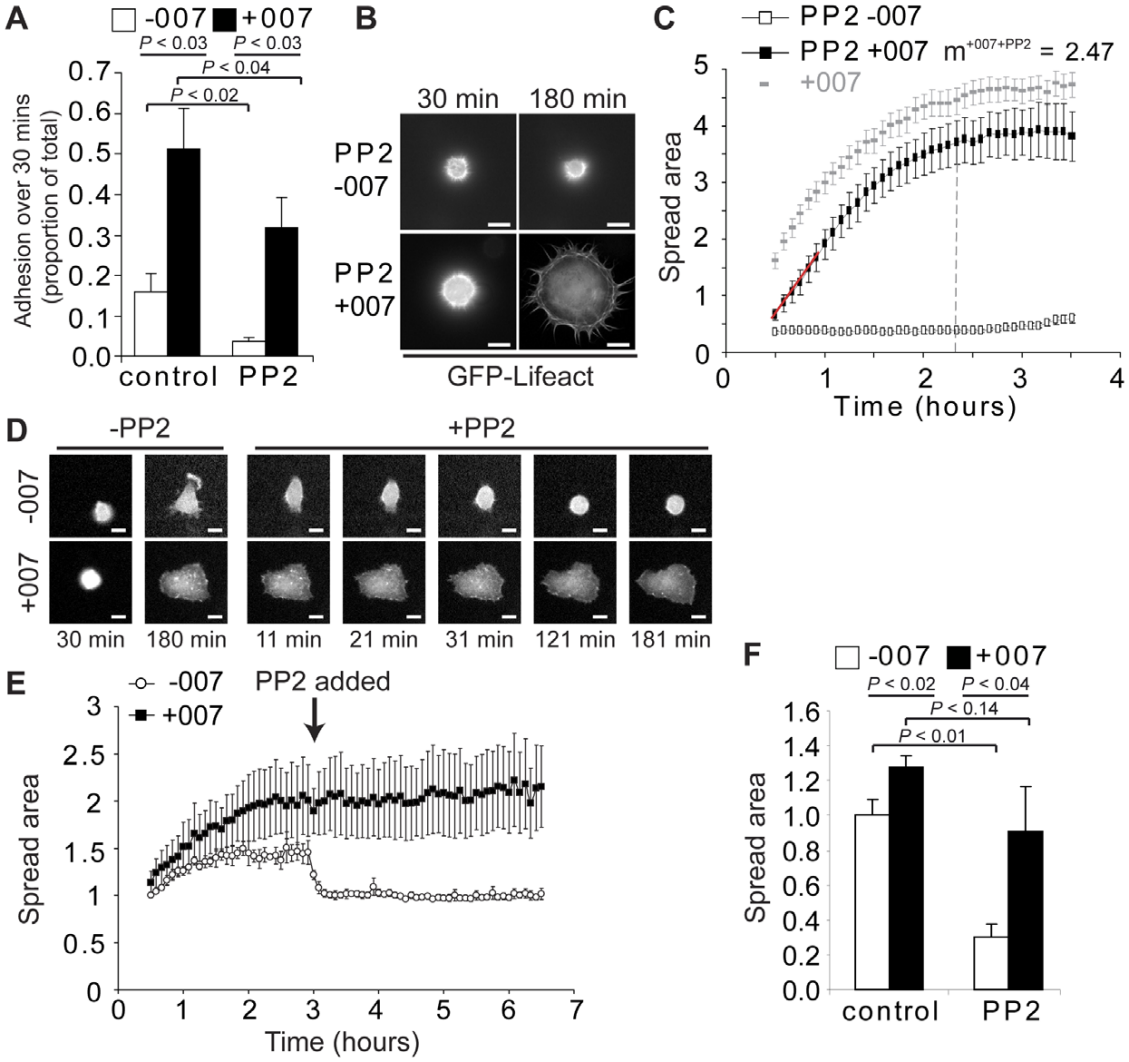
Effects of the Src family kinase inhibitor, PP2, on basal and 007-induced cell spreading. Cells were trypsinised, rolled for 1 hour without or with 20 µM PP2 pre-treatment and plated on fibronectin with or without 007. Adhesion of A549-Epac1 cells after 30 minutes was determined by alkaline phosphatase activity (A). PP2-treated A549-Epac1-GFP-Lifeact cells were plated on fibronectin and imaged over time using the 63×objective. Shown are representative images (B) and quantification of the mean spread area ± the standard error of the means (C) of 16 cells plated without 007 and 15 cells with 100 µM 007 from three separate spreading experiments. Spread area was standardised to the average area of −007 cells shown in Figure 1B at the first frame of imaging (from assays performed independently, but under comparable experimental conditions). The dashed grey curve shows the spreading kinetics of 007-treated cells from Figure 1B. The vertical line indicates the time that cells reached their maximum spread area. The spreading rate was calculated using the gradient (m), in spread area per hour, of the red line shown on the graph. In (D) and (E), A549-Epac1-GFP-Lifeact-expressing cells captured through the 20×objective spread in the presence or absence of 100 µM 007 for 2.5 hours before 20 µM PP2 was added. For quantification (E), the spread area was standardised to the average area of −007 cells at the first time point of imaging. In (F), human umbilical vein endothelial cells were plated on fibronectin for 1 hour in the presence and absence of 100 µM 007 and 20 µM PP2, before being fixed and stained. At least 30 cells per condition were quantified and standardised to the mean area of cells spreading without 007 or PP2. In (A) and (F), graphs show the mean of 5 and 3 experiments, respectively ± the standard error of the means. *P* values were calculated by paired student’s t-tests.

Outside-in activation of Src kinases is crucial for stabilising the cell-ECM adhesion and for promoting the membrane protrusion that initiates cell spreading as cells contact an ECM ligand [4], [10], [12], [14]. To determine if 007 promoted isotropic cell spreading via modulation of the Src kinase signalling pathway, we investigated the effects of the Src kinase inhibitor, PP2, on 007-induced cell adhesion and spreading processes. Pre-treatment of cells with PP2 blocked cell adhesion and prevented cell spreading (Figure 3A, 3B and 3C and Movie S9). However, 007 could still induce adhesion in the presence of PP2 significantly, although it was inhibited compared to the level of cell adhesion observed in the presence of 007 alone (Figure 3A). Quantification of cell spreading revealed that 007- and PP2-treated cells spread to approximately 80% of the size of cells stimulated with only 007, and had a spread area which was 1.6 times that of the basal spreading cells (Figure 1B and Figure 3C and Movie S10). Furthermore, in the presence of 007 and PP2, both the initial rate of cell spreading and the point at which the steady state spread area was approached (140 minutes) matched the spreading kinetics of cells treated with 007 alone (Figure 1B and Figure 3C). These data suggest that both Src- and Rap-mediated cell adhesion pathways contribute to the extent of cell adhesion and spreading observed when 007 is added to cells, but that Src activity is not required for 007 to enhance adhesive processes. When PP2 was added after cells were allowed to spread for three hours either in the presence or absence of 007, unstimulated cells retracted, and rounded-up to resemble their pre-spread state (Figure 3D and 3E and Movie S11). However, cells treated with 007 maintained their existing spread area upon PP2 addition (Figure 3D and 3E and Movie S12). To exclude that Src-independent cell spreading is a peculiarity of the A549 cancer cell line, we investigated the spreading of primary human umbilical vein endothelial cells that contain endogenous Epac1. Spreading of these cells was clearly stimulated after 1 hour in the presence of 007 and PP2, although the basal spreading was inhibited by PP2 (Figure 3F). These data demonstrate that 007-induced Rap1 cell signalling can bypass the need for activating Src kinase activity to induce cell adhesion and spreading.

### Adhesion and Spreading Induced by 007 is Mediated by the Formation of Focal Adhesions that have a Functional Link to the Actin Cytoskeleton

Src activation promotes cells to attach and increase their contact area with their ECM environment by inducing the formation and maturation of integrin-based FAs that are connected to the actin cytoskeleton. These FAs are mechanosensitive, with their size and morphology being altered in response to changes in the contractility of the actomyosin network. Increased actomyosin-induced tension induces larger focal adhesions, and, conversely, a decrease in actomyosin-based tension causes FAs to shrink [10], [62], [63], [64]. We, therefore, investigated if Rap1 could assemble similar integrin-dependent and mechanosensitive adhesion complexes in the absence of the tyrosine phosphorylation steps induced by Src activation.

**Figure 4:**
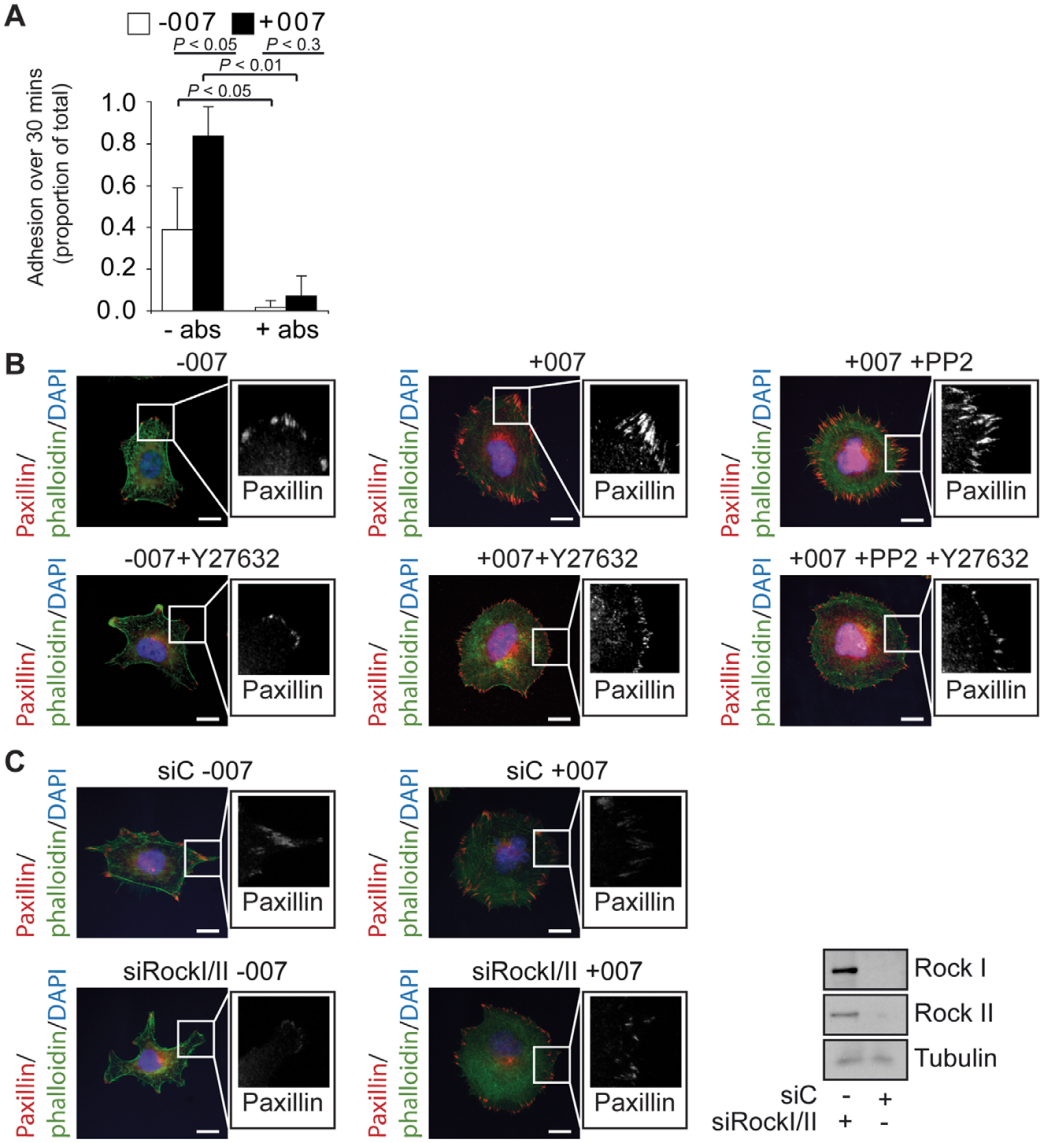
The dependency of 007-induced adhesion and spreading on an integrin-actin link. A549-Epac1 cells were trypsinised, rolled for 1 hour and plated on fibronectin in the presence or absence of 100 µM 007. The effect of integrin inhibiting antibodies (abs) on cell adhesion was determined by alkaline phosphatase activity (A). The graph shows the mean result of three experiments ± the standard error of the means. The *P* values were calculated using a paired student’s t-test. A549-Epac1 cells which had spread for 3 hours in the absence or presence of 100 µM 007, with or without 20 µM PP2 or 10 µM Y27632 (B), or cells which had been transfected with scrambled siRNA (siC) or siRNA against Rock I and Rock II together (siRockI/II) for 48 hours and allowed to spread on fibronectin in the presence or absence of 100 µM 007 (C) were fixed and stained with paxillin, to visualise focal adhesions, phalloidin, to detect F-actin, and DAPI. Enlargements of the paxillin staining from within the regions marked on the merged image are shown alongside. All scale bars represent 10 µm. Western blot analysis to confirm the depletion of Rock I and Rock II is shown alongside.

Firstly, we tested whether the adhesion processes induced by Rap1 were integrin dependent. A549-Epac1 cells were allowed to adhere to fibronectin for 30 minutes in the absence or presence of antibodies which inhibit the function of β1 and β3 integrins in focal adhesions, and the α6 integrin of hemidesmosomes. Both basal and 007-induced adhesion over 30 minutes was inhibited by a mixture of these antibodies, confirming that Rap-induced adhesion requires the activity and function of integrins (Figure 4A). The 007-induced adhesion response was only partially blocked by incubation with the β1-inhibiting antibody alone (data not shown). This is consistent with our previous findings that depletion of the integrin activator, talin, blocked 007-induced spreading in these A549-Epac1 cells [57].

We then investigated the morphology and mechanosensitivity of FAs induced by 007 in cells in which Src was inhibited in order to determine their connection to the actin cytoskeleton. Cells treated with 007 showed FAs around the periphery of the cell, and this was not inhibited by PP2 (Figure 4B). Inhibition of Rock kinases using the inhibitor, Y27632, had no effect on the basal or 007-induced cell spreading response but did decrease the size of adhesions formed under basal conditions or in the presence of 007 or 007+PP2 (Figure 4B). This was also observed with co-depletion Rock I and Rock II (Figure 4C), and indicates that our previous findings that 007-induced cell spreading was inhibited by siRNA depleting only Rock II [57] reflects the differential cellular roles of Rock I and Rock II [65], [66]. Together, these results show that integrin-based, actomyosin-connected and mechanosensitive focal adhesions were induced by Rap1 activity in the presence of the Src inhibitor, PP2.

### 007 does not Induce Tyrosine Phosphorylation of FAK and Paxillin

**Figure 5:**
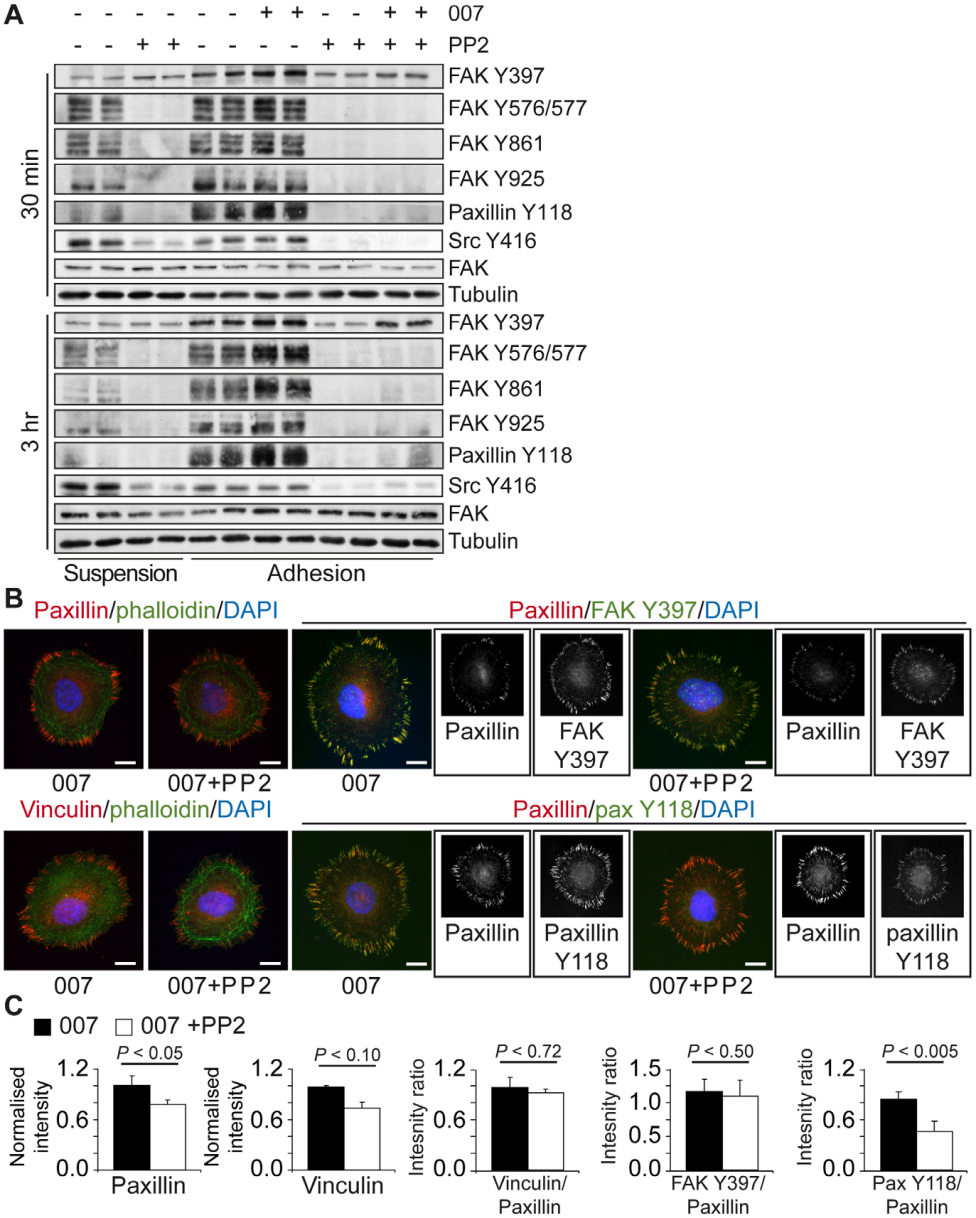
Effect of PP2 on 007-induced focal adhesions. Cells were trypsinised and allowed to roll for 1.5 hours in the absence or presence of 20 µM PP2, before being plated onto fibronectin for 30 minutes or 3 hours with or without 100 µM 007. Cells were then lysed in Laemmli sample buffer and proteins were separated by SDS-PAGE. The levels of phospho-FAK, phospho-Src and phospho-paxillin were determined by western blot (A). A representative of three individual experiments is shown. Rap1-induced focal adhesion composition was investigated by immunofluorescence. Following 3 hours of spreading, cells were fixed and stained for focal adhesions, using anti-paxillin, anti-vinculin, anti-FAK Y397 and anti-paxillin Y118 antibodies, or F-actin, using phalloidin. Representative images are shown in (B), with greyscale images of the single channel fluorescence from the dual stained focal adhesions shown alongside the merged images. The scale bars in the merged images represent 10 µm. (C) The fluorescent intensity of the focal adhesion staining was determined and standardised to the cellular background. Total paxillin or vinculin levels were normalised to the mean intensity of the 007 condition, and the ratio of vinculin levels to paxillin levels present in each condition was calculated. The levels of phospho-FAK and phospho-paxillin were calculated as a ratio with respect to the co-stained intensity of paxillin. The graphs show the average results for 3 experiments (with 10 cells for each condition) ± the standard error of the means. A paired student’s t-test was used to calculate *P* values.

The canonical model of cell adhesion states that adhesion-induced autophosphorylation of FAK pY397 creates a docking site for Src, which then leads to further phosphorylation of FAK on pY576, pY577, pY861 and pY925, as well as the phosphorylation of other adhesion proteins, such as paxillin, which is phosphorylated on pY31 and pY118 [10], [13], [15]. Therefore, we investigated the effect of PP2 and 007 on these phosphorylation events. Pre-treatment of cells with PP2 slightly inhibited the autophosphorylation of FAK (Y397) at the initial stages of adhesion, whilst strongly reducing the Src-regulated phosphorylation of FAK (pY576/577, pY861, pY925) and paxillin (pY118) in A549-Epac1 cells replated and adhered for 30 minutes or 3 hours (Figure 5A). In cells kept in suspension, PP2 acutely promoted FAK autophosphorylation, possibly through a signalling feedback loop to promote cell survival when Src signals were decreased. Stimulation of cells with 007 did not induce the phosphorylation of these proteins in the presence of PP2 (Figure 5A). Therefore, any increase in phospho-paxillin and phospho-FAK observed in response to 007 in the absence of PP2 (comparing lanes 5 and 6 with lanes 7 and 8, Figure 5A) is likely to be an indirect activation of Src through integrin adhesion processes induced by Rap1. From these results, we conclude that activation of Rap1 did not promote compensatory tyrosine phosphorylation of FAK and paxillin, but rather bypassed the need for Src signalling to induce cell adhesion and spreading.

### In the Absence of Src Signalling 007 Still Induces the Formation of Vinculin-containing Focal Adhesions

Phosphorylation of FAK and paxillin is crucial for the assembly of focal adhesions because of their ability to recruit other proteins to the growing adhesion complexes [4], [10], [12]. Vinculin is an important component of adhesions that contributes to reinforcing the link between actin and integrins when FAs are under tension [16], [17], [18], [19], [20], [21]. Previously, the recruitment of vinculin to adhesions has been attributed to Src-driven phosphorylation of paxillin [63]. As 007-induced adhesions in the presence of PP2 are under actomyosin tension, we investigated if vinculin could still be recruited these FAs. We found that adhesions induced by 007 in the presence of PP2 still stained strongly for paxillin, vinculin and FAK pY397 (the auto-phosphorylation site that is upstream of Src recruitment) (Figure 5B). To measure the abundance of proteins specifically localised to FAs, we used quantitative image analysis to measure the fluorescence intensity of FAs relative to the cellular background signal. PP2 reduced the fluorescence intensity levels of paxillin and vinculin in FAs compared to those in FAs of 007-treated cells to about 70% (Figure 5C), showing that FAs were less protein dense in the presence of PP2. However, the reduction in vinculin levels was similar to the reduction in paxillin levels, and the ratio between vinculin and paxillin was not affected by inhibition of Src (Figure 5C). Thus, there was no defect in the ability of vinculin to be recruited to adhesions in the presence of 007 and PP2. Moreover, whereas the ratio of FAK pY397 fluorescence intensity to total paxillin intensity was unaffected by PP2, the ratio of paxillin pY118 to total paxillin fluorescence intensity was significantly inhibited (Figure 5B and 5C). From these data, we conclude that there are multiple mechanisms through which proteins can associate together at sites of adhesions. The current models of FA assembly state that tyrosine phosphorylation of adhesion components by Src is critical for the assembly of integrins, paxillin and other components, such as vinculin, into focal adhesions. Our data demonstrate that Rap1 can induce an alternative FA assembly pathway.

### Auto-phosphorylation of FAK is not Required for 007-induced Adhesion, Spreading and Focal Adhesion Formation

**Figure 6:**
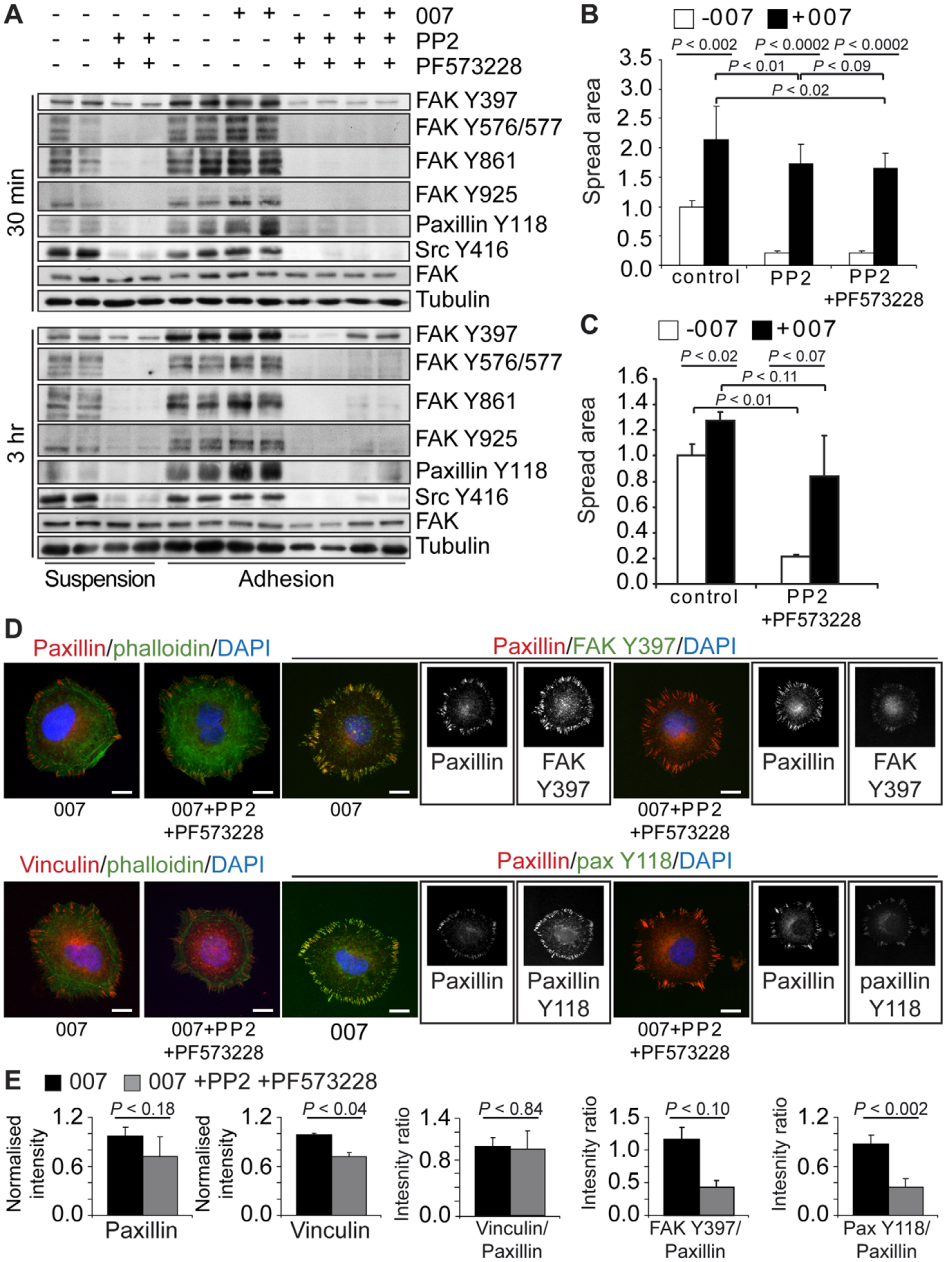
Effect of inhibiting FAK and Src activation on 007-induced cell spreading and focal adhesions. Cells were trypsinised and allowed to roll for 1.5 hours in the absence or presence of 20_µM PP2 and 1 µM PF573228 before analysis. In (A), A549-Epac1 cells were plated onto fibronectin for 30 minutes or 3 hours with or without 100 µM 007 and were then lysed in Laemmli sample buffer. Proteins were separated by SDS-PAGE and phospho-FAK, phospho-Src and phospho-paxillin levels were determined by western blot. A representative of 3 individual experiments is shown. To analyse spreading, A549-Epac1 cells were replated and allowed to spread for 3 hours (B) or human umbilical vein endothelial cells were allowed to spread for 1 hour (C) before being fixed and stained. For each experiment, the spread area of at least 30 cells per condition was quantified and standardised to the mean area of cells spreading without 007 or inhibitors. The graphs in (B) and (C) show the mean of 6 or 3 experiments, respectively ± the standard error of the means. Focal adhesion composition after 3 hours of spreading was determined by immunofluorescence using antibodies against paxillin, FAK Y397, paxillin Y118 and vinculin (D). Representative cells are shown, with greyscale images of the single channel fluorescence from the dual stained focal adhesions shown alongside the merged images. The scale bars represent 10 µm. The fluorescent intensity of the focal adhesion staining was determined and standardised to the cellular background (E). Total paxillin or vinculin levels were normalised to the intensity of the 007 condition, and the ratio of vinculin to paxillin present in each condition was calculated. Phospho-FAK and phospho-paxillin levels were calculated as ratios of the fluorescent intensity of the co-stained paxillin. The average results for 3 experiments are shown (with 10 cells for each condition) ± the standard error of the means. *P* values were calculated using paired student’s t-tests.

As treatment with the Src inhibitor did not block the kinase activity and autophosphorylation of FAK at pY397, we pre-treated cells with both PP2 and the FAK inhibitor, PF573228, to investigate if FAK signalling to proteins other than Src and paxillin was needed for Rap1-induced FA formation. Combined pre-treatment of cells with PP2 and PF573228 strongly inhibited the adhesion-induced phosphorylation of FAK and paxillin observed after 30 minutes and 3 hours of adhesion, and 007 did not induce either the autophosphorylation of FAK or the phosphorylation of Src substrates substantially (Figure 6A). Pre-treatment with PP2 and PF573228 blocked the basal spreading of cells, but the spreading induced with 007 was significant in both A549-Epac1 cells (Figure 6B and 6D) and in human umbilical vein endothelial cells (Figure 6C). As judged by western blot, after three hours of attachment and spreading, pY397 FAK levels in cells treated with 007 increased to levels approaching those found in cells in suspension in the absence of the inhibitor (Figure 6A). To examine the importance of FAK in 007-induced spreading further, we depleted FAK from cells using shRNA and siRNAs targeting FAK (Figure S2). FAK levels were depleted most effectively by transfection of stable FAK-knockdown cells with siRNA against FAK (Figure S2A), and under this condition, FAK staining of FAs was most strongly decreased, although residual staining still remained (Figure S2B). FAK depletion of cells stimulated the basal spreading of cells, and did not block the 007 spreading response (Figure S2C). Moreover, treatment of FAK depleted cells with PP2 and PF573228 blocked the basal spreading response, but 007 could still induce significant spreading of the cells (Figure S2C). Together, these data strongly support the conclusion that FAK levels and activity are not critical for 007-induced spreading.

The A549-Epac1 cells spreading in response to 007 in the presence of PP2 and PF573228 showed a morphology closely resembling that of the cells spreading in the presence of 007 and PP2, but focal adhesions did not stain for FAK pY397 and paxillin pY118 (Figure 6D). Quantification of the fluorescence intensities of these components confirmed that, compared to control cells, the ratio between FAK pY397 and paxillin or paxillin pY118 and paxillin was strongly reduced by PP2 and PF573228 (Figure 6E). Nevertheless, as observed with PP2 treatment alone, we found that the adhesions induced by 007 in the presence of PP2 and PF573228 stained for vinculin (Figure 6D) and that the ratio in the fluorescence intensity of vinculin and paxillin remained the same as in cells treated with 007 alone (Figure 6E). This shows that Rap1 activation can drive recruitment of vinculin to FAs even during combined inhibition of FAK and Src activity.

## Discussion

In this study, we developed image analysis tools for the automated and quantitative analysis of cell spreading. For the first time, we have been able to make a detailed analysis of how activation of Rap1 modulates the kinetics and morphology of cells spreading over a fibronectin matrix. We observed that, under basal conditions, A549-Epac1 cells exhibited an anisotropic mode of spreading, where both an increase in cell spread area and a steady state of spread area was accompanied by extensive remodelling of the overall shape of the spread area of cells, mediated through continuous protrusion and retraction of the cell periphery. This anisotropic mode of spreading gave rise to the angular morphology of A549-Epac1 cells observed in fixed samples [57]. Addition of 007, to activate endogenous Rap proteins specifically via the exchange factor Epac1, promoted a faster rate of cell spreading, induced cells to spread isotropically and reduced the remodelling of the spread area which occurred under basal conditions.

A factor that controls whether cells spread isotropically or anisotropically is the availability of ECM ligand sites to which cells can attach and make protrusions [4]. Lower ECM concentrations promote anisotropic spreading, as cells have to “search” for ligand-dense sites which activate Src sufficiently to induce the formation of stable attachments [4]. Our results show that Rap1 activation can induce isotropic spreading on an ECM substratum that otherwise drives anisotropic spreading. This indicates that Rap1 can promote and stabilise functional adhesion complexes at ligand sites that integrins may be able to attach to, but may not be sufficient to activate Src-driven protrusion. The ability of Rap1 to induce adhesion and spreading in the presence of PP2, even though outside-in signalling and the basal adhesion of A549-Epac1 cells were completely blocked, further supports this conclusion. Through the ability to induce adhesion, spreading and cell morphology changes by mechanisms that do not rely on the canonical ECM-derived signals, Rap1 may influence many stages of tissue development, including stem cell differentiation which is, in part, determined by the constraints induced by the extracellular matrix microenvironment [67], [68], [69].

One explanation for the rescue of spreading in the presence of PP2 is that, through the GEF, C3G, Rap1 functions downstream of Src, and, by stimulating Rap1, the next step in the Src pathway is being re-activated [52]. However, our data show that the canonical phosphorylation sites in the FAK-Src-Paxillin module that were blocked by the inhibitors, PP2 and PF573228, were not rescued by Epac1-induced Rap1 activation. These data demonstrate that Rap1 does not regulate the FAK-Src signalling pathway and that the Src pathway is not truly reconstituted by Rap1 activation. Furthermore, as activation of Rap1 via C3G is reported to be mediated, in part, by actomyosin-induced force [53], our findings that Epac1-induced Rap1 activation can promote force-dependent FAs indicate that activating Rap1 is not simply reconstituting the Src pathway one-step down. Rather, our results place Rap1 regulation of adhesion and spreading in a parallel, or bypass, pathway from the FAK-Src-signalling module.

We have shown that Rap1 activation creates an ECM-integrin-actomyosin link, and focal adhesions that were responsive to changes in actomyosin contractility, in order to promote adhesion and spreading. It is, therefore, likely that the mechanisms by which Rap1 initiates spreading and FA formation in the presence of PP2 are analogous to the processes by which the FAK-Src module controls adhesion. It is well characterised that the FAK-Src signalling module induces a transient reduction of RhoA activity to promote cell spreading, followed by an induction of actomyosin-induced tension which induces the maturation of FAs [4], [10], [12]. Therefore, as Rap1-initiated spreading in the presence of PP2, our data indicate that Rap1 itself could lower actomyosin-induced tension. Furthermore, as 007-induced focal adhesions were modulated by actomyosin contractility, it suggests that Rap may mediate a temporal and localised regulation of RhoA activity, similar to that which is instigated during Src-mediated spreading [4], [10], [12]. Although Rap1 is implicated in modulating actomyosin tension, it is currently unclear which Rap effectors would regulate RhoA in our cell system, as we previously found that siRNA against Arap3, Krit1, or RA-RhoGAP/ARHGAP20 did not alter Rap-induced cell spreading of A549-Epac1 cells [57].

Our data implicate Rap1 activity as one of the mediators of force-dependent strengthening and maturation of FAs, a process that previously has largely been attributed to the FAK-Src-Paxillin cascade [63]. Indeed, we found that the relative level of vinculin in Rap1-induced FAs was not altered upon the inhibition of the FAK-Src module although paxillin phosphorylation was strongly inhibited. In laser tweezer experiments using fibronectin-coated beads, mechanical force in the absence of Src kinase activity has been demonstrated to recruit vinculin to bead-cell focal complexes [16]. Thus, in Rap1 activated and Src-inhibited cells, we created a similar situation at FAs, and demonstrated that Rap1 activation permitted the recruitment of vinculin to adhesions. As adhesions induced by Rap1 are mechanosensitive, the mechanism by which Rap1 activation recruits vinculin to adhesions may be primarily via stretch-dependent unfolding of Talin [16], [20], [70], [71], [72], [73]. This requires further investigation, however, our model system that combines inhibition of Src with active Rap1, will be a valuable tool for the characterisation of FAK-Src independent mechano-regulation of integrin adhesions.

Together, our data demonstrate that activation of Rap1 induces a functional ECM-integrin-actomyosin link that promotes adhesion and spreading, but which does not depend on the activity of the FAK-Src signalling module. Thus, Rap1 induced adhesion and spreading in A549-Epac1 cells shows similarities to Src-induced processes, but we propose that they are driven by parallel pathways. By regulating the ECM-integrin-actin link by distinct molecular mechanisms from the FAK-Src-paxillin cascade, a localised activation of Rap1 in cells within tissues may act to reinforce the contacts between cells and their extracellular environment. Indeed, the regulation of Rap1 activity has recently been implicated as being critical for preserving the attachment of neural stem cells to their niche [74]. Therefore, activation of Rap1 may play a vital and significant role in modulating the physiological interaction between cells and their extracellular matrix environment to contribute to the structural maintenance and integrity of tissues.

## Supporting Information

Figure S1 **Quantification of cell spreading.** Cell protrusion and retraction were quantified by detecting areas of pixel gain or loss between successive frames of time-lapse movies (A). Cells edges (shown in red) were detected using local watershed segmentation upon smoothing and the images binarised. The binarised image at time point, t, (I(t), middle panel), was subtracted from the binarised image of the subsequent frame of the movie (I(t+1), left panel) to determine unchanged regions (grey), lost areas (black) or pixels gained (white) between the two frames. Sizes of areas lost or gained were determined, and expressed as fractions of the total area of the cell in frame I(t+1). The circle ratio was used to evaluate cell shape at each frame of a time-lapse movie (B). The area of a circle (dashed circle) with a radius equalling the length from the centre of the cell to the most distant pixel of the detected cell edge (solid red line) was determined. The ratio between the area of the cell and the circle was then calculated. Shape changes were analysed by determining the variance of the lengths from the centre of the cell to each pixel detected on the periphery. In (C), the black lines mark the shortest and longest lengths from the centre of the cell to the periphery for two consecutive frames of a time-lapse movie of a cell spreading without 007 (top panel) and with 007 (bottom panel) to illustrate how these may change over time. The normalised standard deviation for all the points of the cell periphery (σ_n_) is written underneath the cell. The difference of the variance (Δσ_n_) was calculated by subtracting the σ_n_ value for time point t+1 from the σ_n_ at time point t. Cells displaying dynamic shape changes have a higher Δσ_n_ over time.
(TIF)

Figure S2 **Effect of FAK depletion on cell spreading.** A549-Epac1 cells were transduced with either a control (shC002) lentiviral short hairpin, or one targeting FAK (shFAK), and stable expression of the constructs were selected by adding puromycin selection to the growth media. These cells were then subjected to transient transfection of control (siC) or siRNA targeting FAK (siFAK). Depletion of FAK under different conditions was determined by western blot (A). A549-Epac1 cells treated with control and FAK-targeting short hairpins and siRNA were trypsinised and allowed to roll for 1 hour in the absence or presence of 20 µM PP2 and 1 µM PF573228 before being plated onto fibronectin with or without 100 µM 007. They were allowed to spread for 3 hours before being fixed and stained. The FAK content of focal adhesions after 3 hours of spreading was determined by immunofluorescence (B). Representative images of cells treated with 100 µM 007 are shown, with greyscale images of the anti-FAK focal adhesion staining shown alongside the merged images. The scale bars represent 10 µm. Images of at least 30 cells per condition per experiment were captured, and ImageJ was used to quantify the spread area of the cells (C). For each experiment, the spread area of cells in each condition was standardised to the mean area of cells treated with control shRNA and siRNA, spreading without 007 or inhibitors. The graph in (C) shows the average of 2 experiments ± the range of the means. The *P* values were calculated using a paired student’s t-test.
(TIF)

Movie S1 **Representative time-lapse movie of basal cell spreading.** A549-Epac1 cells expressing GFP-Lifeact were trypsinised and allowed to roll for 1 hour before plating out onto fibronectin. Filming began approximately 30 minutes after cells were first applied to the fibronectin. Images were captured every 5 minutes through the 63×objective, and the movie rate is 7 frames per second. The scale bar represents 10 µm.
(MOV)

Movie S2 **Representative time-lapse movie of 007-induced cell spreading.** A549-Epac1 cells expressing GFP-Lifeact were trypsinised and allowed to roll for 1 hour before plating out onto fibronectin in the presence of 100 µM 007. Filming began approximately 30 minutes after cells were first applied to the fibronectin. Images were captured every 5 minutes through the 63×objective, and the movie rate is 7 frames per second. The scale bar represents 10 µm.
(MOV)

Movie S3 **Representative automated detection of basal cell spreading.** A549-Epac1 cells expressing GFP-Lifeact were trypsinised and allowed to roll for 1 hour before plating out onto fibronectin. Filming began approximately 30 minutes after cells were first applied to the fibronectin. Images were captured every 5 minutes through the 63×objective, and the movie rate is 7 frames per second. The scale bar represents 10 µm. In each frame, the cell was detected using local watershed segmentation upon smoothing (the detected cell edge is shown in red).
(MOV)

Movie S4 **Representative automated detection of 007-induced cell spreading.** A549-Epac1 cells expressing GFP-Lifeact were trypsinised and allowed to roll for 1 hour before plating out onto fibronectin in the presence of 100 µM 007. Filming began approximately 30 minutes after cells were first applied to the fibronectin. Images were captured every 5 minutes through the 63×objective, and the movie rate is 7 frames per second. The scale bar represents 10 µm. Detection of cells was performed by local watershed segmentation upon smoothing (the detected cell edge is shown in red).
(MOV)

Movie S5 **Time-lapse movie of basal cell spreading of cells treated with scrambled siRNA.** A549-Epac1 expressing GFP-Lifeact cells were treated with scrambled siRNA for 48 hours before being replated onto fibronectin (without 007). Images of multiple cells were captured through the 20×objective every 5 minutes for the following 3 hours. The movie rate is 7 frames per second. The scale bar represents 10 µm.
(MOV)

Movie S6 **Time-lapse movie of basal spreading of Rap1A- and Rap1B-depleted cells.** A549-Epac1 cells expressing GFP-Lifeact were co-transfected with siRNA against Rap1A and Rap1B for 48 hours before being replated onto fibronectin (without 007). Images of multiple cells were captured through the 20×objective every 5 minutes for the following 3 hours. The movie rate is 7 frames per second. The scale bar represents 10 µm.
(MOV)

Movie S7 **Time-lapse movie of cells treated with scrambled siRNA spreading in the presence of 007.** A549-Epac1 expressing GFP-Lifeact cells were treated with scrambled siRNA for 48 hours before being replated onto fibronectin with 100 µM 007. Images of multiple cells were captured through the 20×objective every 5 minutes for the following 3 hours. The movie rate is 7 frames per second. The scale bar represents 10 µm.
(MOV)

Movie S8 **Time-lapse movie of Rap1A- and Rap1B-depleted cells spreading in the presence of 007.** A549-Epac1 cells expressing GFP-Lifeact were co-transfected with siRNA against Rap1A and Rap1B for 48 hours before being replated onto fibronectin with 100 µM 007. Images of multiple cells were captured through the 20×objective every 5 minutes for the following 3 hours. The movie rate is 7 frames per second. The scale bar represents 10 µm.
(MOV)

Movie S9 **Time-lapse movie of basal cell spreading in the presence of PP2.** A549-Epac1 cells expressing GFP-Lifeact were trypsinised and allowed to roll for 1 hour in the presence of 20_µM PP2 before plating out onto fibronectin. Filming began approximately 30 minutes after cells were first applied to the fibronectin. Images were captured every 5 minutes through the 63×objective, and the movie rate is 7 frames per second. The scale bar represents 10 µm.
(MOV)

Movie S10 **Time-lapse movie of 007-induced cell spreading in the presence of PP2.** A549-Epac1 cells expressing GFP-Lifeact were trypsinised and allowed to roll for 1 hour in the presence of 20_µM PP2 before plating out onto fibronectin in the presence of 100 µM 007. Filming began approximately 30 minutes after cells were first applied to the fibronectin. Images were captured every 5 minutes through the 63×objective, and the movie rate is 7 frames per second. The scale bar represents 10 µm.
(MOV)

Movie S11 **Time-lapse movie showing the effect of addition of PP2 to cells spreading under basal conditions.** GFP-Lifeact-expressing A549-Epac1 cells were trypsinised and kept in suspension for 1 hour before being plated out onto fibronectin. Images were captured for 2.5 hours from approximately 30 minutes after cells were plated. Then 20 µM PP2 was added to the cells (blank frame) and the response of cells was followed for a further 3 hours. Images were captured every 5 minutes through the 20×objective, and the movie rate is 7 frames per second. The scale bar represents 10 µm.
(MOV)

Movie S12 **Time-lapse movie showing the effect of addition of PP2 to cells spreading in the presence of 007.** GFP-Lifeact-expressing A549-Epac1 cells were trypsinised and kept in suspension for 1 hour before being plated out onto fibronectin in the presence of 100 µM 007. Images were captured for 2.5 hours from approximately 30 minutes after cells were plated. Then 20 µM PP2 was added to the cells (blank frame) and the response of cells was followed for a further 3 hours. Images were captured every 5 minutes through the 20×objective, and the movie rate is 7 frames per second. The scale bar represents 10 µm.
(MOV)
