# Supplementary figures and images for: Rap1 Can Bypass the FAK-Src-Paxillin Cascade to Induce Cell Spreading and Focal Adhesion Formation
Source: PLoS One. 2012 Nov 27;7(11):e50072. doi: 10.1371/journal.pone.0050072 (PMC3507925; doi:10.1371/journal.pone.0050072)

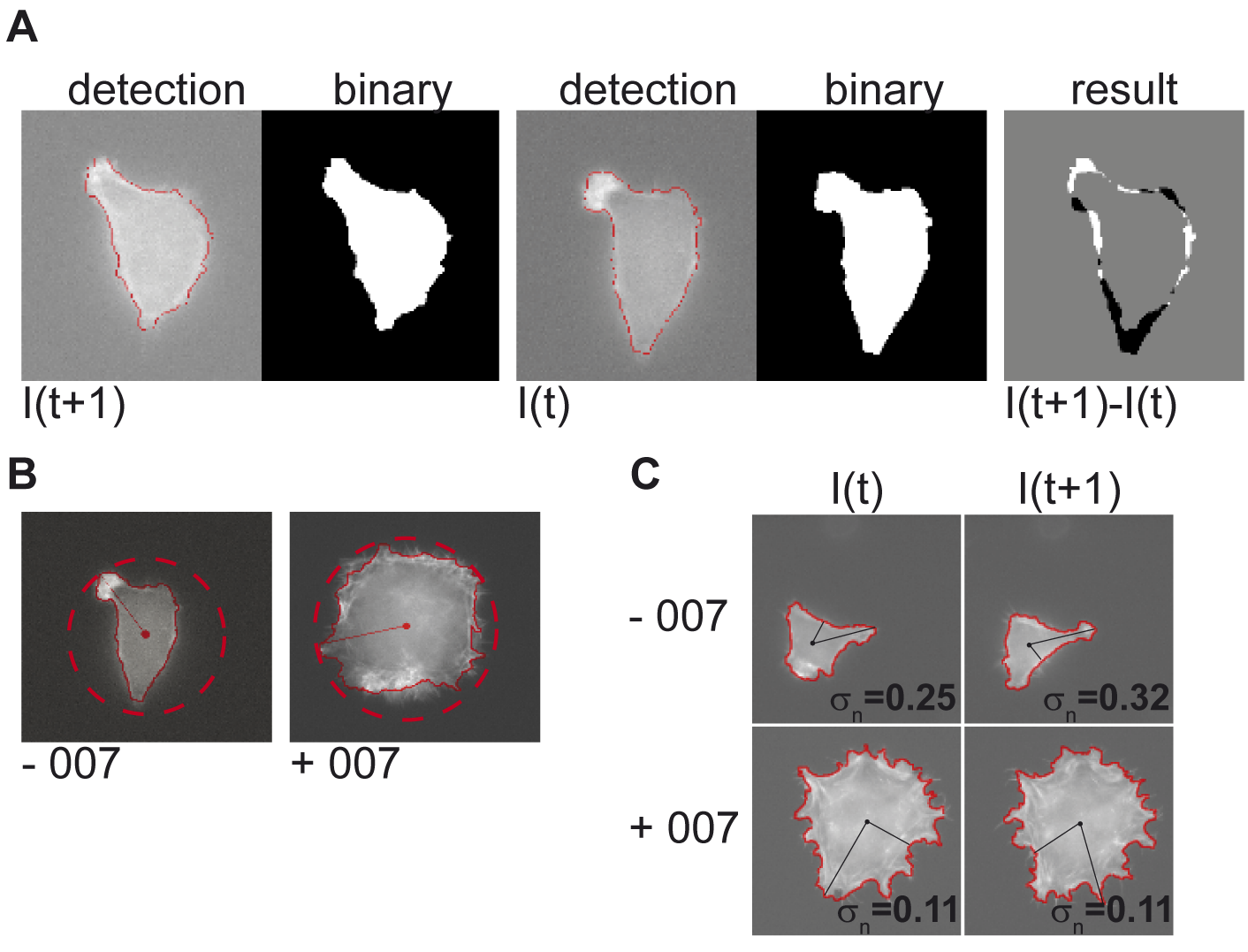

Supplement: Figure S1 — Quantification of cell spreading. Cell protrusion and retraction were quantified by detecting areas of pixel gain or loss between successive frames of time-lapse movies (A). Cells edges (shown in red) were detected using local watershed segmentation upon smoothing and the images binarised. The binarised image at time point, t, (I(t), middle panel), was subtracted from the binarised image of the subsequent frame of the movie (I(t+1), left panel) to determine unchanged regions (grey), lost areas (black) or pixels gained (white) between the two frames. Sizes of areas lost or gained were determined, and expressed as fractions of the total area of the cell in frame I(t+1). The circle ratio was used to evaluate cell shape at each frame of a time-lapse movie (B). The area of a circle (dashed circle) with a radius equalling the length from the centre of the cell to the most distant pixel of the detected cell edge (solid red line) was determined. The ratio between the area of the cell and the circle was then calculated. Shape changes were analysed by determining the variance of the lengths from the centre of the cell to each pixel detected on the periphery. In (C), the black lines mark the shortest and longest lengths from the centre of the cell to the periphery for two consecutive frames of a time-lapse movie of a cell spreading without 007 (top panel) and with 007 (bottom panel) to illustrate how these may change over time. The normalised standard deviation for all the points of the cell periphery (σn) is written underneath the cell. The difference of the variance (Δσn) was calculated by subtracting the σn value for time point t+1 from the σn at time point t. Cells displaying dynamic shape changes have a higher Δσn over time. (TIF) [file pone.0050072.s001.tif]

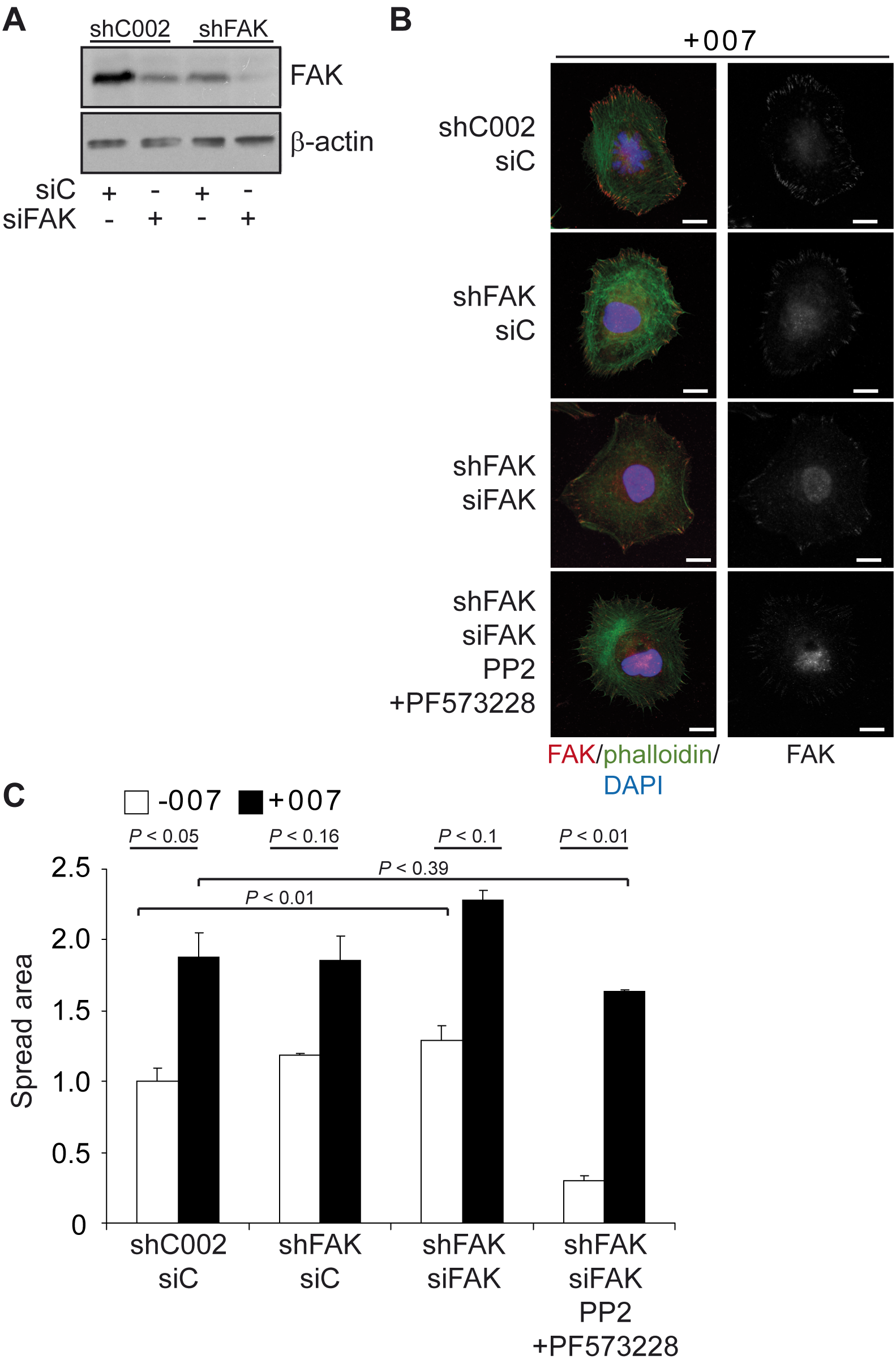

Supplement: Figure S2 — Effect of FAK depletion on cell spreading. A549-Epac1 cells were transduced with either a control (shC002) lentiviral short hairpin, or one targeting FAK (shFAK), and stable expression of the constructs were selected by adding puromycin selection to the growth media. These cells were then subjected to transient transfection of control (siC) or siRNA targeting FAK (siFAK). Depletion of FAK under different conditions was determined by western blot (A). A549-Epac1 cells treated with control and FAK-targeting short hairpins and siRNA were trypsinised and allowed to roll for 1 hour in the absence or presence of 20 µM PP2 and 1 µM PF573228 before being plated onto fibronectin with or without 100 µM 007. They were allowed to spread for 3 hours before being fixed and stained. The FAK content of focal adhesions after 3 hours of spreading was determined by immunofluorescence (B). Representative images of cells treated with 100 µM 007 are shown, with greyscale images of the anti-FAK focal adhesion staining shown alongside the merged images. The scale bars represent 10 µm. Images of at least 30 cells per condition per experiment were captured, and ImageJ was used to quantify the spread area of the cells (C). For each experiment, the spread area of cells in each condition was standardised to the mean area of cells treated with control shRNA and siRNA, spreading without 007 or inhibitors. The graph in (C) shows the average of 2 experiments ± the range of the means. The P values were calculated using a paired student’s t-test. (TIF) [file pone.0050072.s002.tif]
